# Sugarcane Serine Peptidase Inhibitors, Serine Peptidases, and Clp Protease System Subunits Associated with Sugarcane Borer (*Diatraea saccharalis*) Herbivory and Wounding

**DOI:** 10.3390/ijms17091444

**Published:** 2016-09-01

**Authors:** Ane H. Medeiros, Fabiana B. Mingossi, Renata O. Dias, Flávia P. Franco, Renato Vicentini, Marcia O. Mello, Daniel S. Moura, Marcio C. Silva-Filho

**Affiliations:** 1 Departamento de Ciências da Natureza, Matemática e Educação, Universidade Federal de São Carlos, Araras, 13600-970 São Paulo, Brazil; anehm@cca.ufscar.br; 2 Departamento de Genética, Escola Superior de Agricultura Luiz de Queiroz, Universidade de São Paulo, Piracicaba, 13418-260 São Paulo, Brazil; fabiana.mingossi@gmail.com (F.B.M.); diasrdo@gmail.com (R.O.D.); flavia.esalq@gmail.com (F.P.F.); marcia.o.jose@gmail.com (M.O.M.); 3 Systems Biology Laboratory, Center for Molecular Biology and Genetic Engineering, State University of Campinas, Campinas, 13083-970 São Paulo, Brazil; shinapes@unicamp.br; 4 Monsanto do Brasil, Campinas, 13069-380 São Paulo, Brazil; 5 Departamento de Ciências Biológicas, Escola Superior de Agricultura Luiz de Queiroz, Universidade de São Paulo, Piracicaba, 13400-918 São Paulo, Brazil; danielmoura@usp.br

**Keywords:** macroarray, sugarcane, *Diatraea saccharalis*, serine peptidase inhibitors, serine peptidase, Clp protease system, induced resistance, plant–insect interaction

## Abstract

Sugarcane’s (*Saccharum* spp.) response to *Diatraea saccharalis* (F.) (Lepidoptera: (Crambidae) herbivory was investigated using a macroarray spotted with 248 sugarcane Expressed Sequence Tags (ESTs) encoding serine peptidase inhibitors, serine peptidases. and Clp protease system subunits. Our results showed that after nine hours of herbivory, 13 sugarcane genes were upregulated and nine were downregulated. Among the upregulated genes, nine were similar to serine peptidase inhibitors and four were similar to Bowman-Birk Inhibitors (BBIs). Phylogenetic analysis revealed that these sequences belong to a phylogenetic group of sugarcane BBIs that are potentially involved in plant defense against insect predation. The remaining four upregulated genes included serine peptidases and one homolog to the *Arabidopsis* AAA+ chaperone subunit ClpD, which is a member of the Clp protease system. Among the downregulated genes, five were homologous to serine peptidases and four were homologous to *Arabidopsis* Clp subunits (three homologous to Clp AAA+ chaperones and one to a ClpP-related ClpR subunit). Although the roles of serine peptidase inhibitors in plant defenses against herbivory have been extensively investigated, the roles of plant serine peptidases and the Clp protease system represent a new and underexplored field of study. The up- and downregulated *D. saccharalis* genes presented in this study may be candidate genes for the further investigation of the sugarcane response to herbivory.

## 1. Introduction

The sugarcane borer *Diatraea saccharalis* (F.) (Lepidoptera: Crambidae) is the most significant pest of sugarcane, with a wide distribution in Brazilian fields [1]. Sugarcane borer larvae create a vertical tunnel in the stem that becomes the primary route for microorganism entry [2]. The fungi that cause stem rot, *Colletotrichum falcatum* (Went) and *Fusarium verticillioides*, are commonly found in the tunnels produced by *D. saccharalis*.

Plants recognize insect feeding via chemical interactions between self-molecules that may be modified by the insect [3] or via insect molecules [4,5]. This chemical interaction triggers a signaling cascade that causes the expression of defense-associated genes [6,7,8,9].

Plant peptidase inhibitors have been shown to inhibit digestive peptidases in herbivorous insects [10,11,12]. The induction and accumulation of peptidase inhibitors (PIs) after mechanical or insect wounding have been reported in several plant families and are considered part of the plant´s natural defense system against herbivory [10,13].

Bowman–Birk peptidase inhibitors were first isolated and characterized in soybean seeds [14,15] and were subsequently found in other leguminous plants [16,17] and in the Poaceae [18]. These proteins are associated with endogenous seed peptidases regulation, sulfur amino acids storage, and plant defense against pathogens and insects [19]. Their anti-nutritive function is derived from their formation of stable complexes with the catalytic sites of peptidases, blocking degradation and ingestion of amino acids from the insect diet [20,21].

Other than their involvement in responses to other types of stress, and in contrast to peptidase inhibitors, little is known about plant cytoplasmic and intra-plastid serine peptidases and their roles in defense against herbivory [22,23]. These peptidases have a housekeeping role in plants, releasing amino acids for recycling and eliminating non-functional proteins. However, peptidases have also been shown to play important roles in plant defense, acting in pathogen and pest recognition and in induction of effective defense responses [24].

Studies involving other plant species have elucidated the roles of various peptidases in plant defense; for example, in tomatoes, a serine carboxypeptidase was induced by wounding, systemin, and methyl jasmonate treatment [25]. Subtilisins have also been implicated in plant defense against herbivores [26,27]. There is evidence for a subtilase that is involved in systemin processing [28]. In tomatoes, systemin is synthesized in the form of a precursor, and following mechanical or herbivore injury, pro-systemin is processed and translocated throughout the plant, triggering a signaling cascade that ultimately activates defense genes such as proteinase inhibitors [29,30].

Non-serine peptidases have also been shown to be involved in resistance: Mir1-CP, a cysteine peptidase identified in maize *S. frugiperda*-resistant lines, is rapidly induced when plants areinjured [31,32]. The authors investigated why *S. frugiperda* larvae fed with maize leaves experienced diminished growth and found that this peptidase damages the peritrophic matrix of not only *S. frugiperda* but of various insects of the Lepidoptera order [33]. Leucine aminopeptidase A (LapA) is a late wound-response gene of tomato (*Solanum lycopersicum*) that accumulates after mechanical, insect and pathogen wounding [34,35,36]. Working in concert with LapA, arginase and threonine deaminase play roles in plant defense against herbivores as well [35,37,38].

Techniques such as macroarrays, subtractive libraries, AFLP-cDNA (Amplified Fragment Length Polymorphism-cDNA) display, and differential display RT-PCR (Reverse Transcriptase Polymerase Chain Reaction) have enabled researchers to study changes in the transcriptome that are elicited in response to herbivory and wounding [39]. In contrast to research in other more widespread crops, progress in the field of sugarcane genomics has been slow. Genetic analyses are difficult in sugarcane due to its large and complex polyploid genome and the lack of sufficiently informative gene-tagged markers [40]. However, significant progress has been noted after the development of tools such as collections of expressed sequence tags (ESTs). Large EST collections have been made available [41,42,43,44,45] and have consequently renewed interest in sugarcane genetics [46]. Currently, the NCBI (National Center for Biotechnology Information) database lists more than 20,000 ESTs from the *Saccharum officinarum* complex (cultivated sugarcanes), and researchers have used these sequences to identify putative genes for the improvement of sugarcane field performance [47,48,49]. Using microarrays containing sugarcane ESTs, Rocha et al. [50] identified several sugarcane methyl jasmonate- and herbivore-responsive genes.

In silico analyses have shown that sugarcane possesses a set of conserved peptidase inhibitors that may also be involved in defense [19,48]. Initiatives to increase sugarcane borer resistance using traditional breeding and genetic engineering are needed. A better understanding of the sugarcane responses triggered by *D. saccharalis* feeding and wounding via the identification and characterization of genes directly involved in such responses may represent a means to improve sugarcane resistance. Those genes could also be used as molecular markers for insect resistance in traditional breeding programs.

In this study, we custom-made a macroarray containing 248 genes, including peptidase inhibitors, serine peptidases, and Clp protease system subunits, from the sugarcane EST collection. We have identified 10 peptidase inhibitors, seven peptidases, and five Clp subunits that are differentially expressed in sugarcane above-ground tissues in response to *D. saccharalis* feeding at an early time point.

## 2. Results

### 2.1. Macroarray Hybridization

To obtain information regarding the specific roles that peptidase inhibitors and peptidases may play in the sugarcane defense response against herbivores and wounding, a custom-made cDNA macroarray was constructed by spotting 248 selected ESTs on filter membranes (Table S1). The filter membranes were probed with ^33^P cDNA populations derived from RNA extracted from the leaves of undisturbed sugarcane plants (0 and 9 h time points) and from the leaves of plants attacked by *D. saccharalis* (9 h time point). Probed membranes were effective for the identification of differentially expressed ESTs as exemplified by SacMPI-like1 (EST spots highlighted with a square in Supplementary Material Figure S1A–C) and SacBBI1 (EST spots highlighted with a circle in Supplementary Material Figure S1A–C). Both SacMPI-like1 and SacBBI1 exhibited stronger signals when probed with ^33^P cDNA populations derived from the leaves of sugarcane plants attacked by *D. saccharalis* than when they were probed with populations derived from the leaves of undisturbed plants.

Of the 248 genes represented in the macroarrays, 22 presented consistent and reproducible expression in at least two hybridizations out of six possible comparisons (refer to Materials and Methods for more information regarding membrane randomization). Thirteen upregulated and nine downregulated genes were validated by quantitative real-time PCR; they are listed in Table 1 and Table 2.

### 2.2. Validation of D. saccharalis-Inducible Genes

To validate the expression of those genes upregulated after *D. saccharalis* feeding, we performed another set of biological experiments and included one more treatment: mechanical wounding. It was been shown that wounding, either by insects or mechanically, induces a general wounding response in plants [22,52,53,54]. In addition to that, insect wounding provokes a tailored response, specifically induced by some plant components released by insect feeding [3] or present in the insect saliva [53,55]. The objective was to compare the level of transcripts of 10 selected genes, after mechanical wounding and wounding by *D. saccharalis*.

#### 2.2.1. Sugarcane Bowman-Birk Inhibitor (SacBBI) Genes

**Figure 1:**
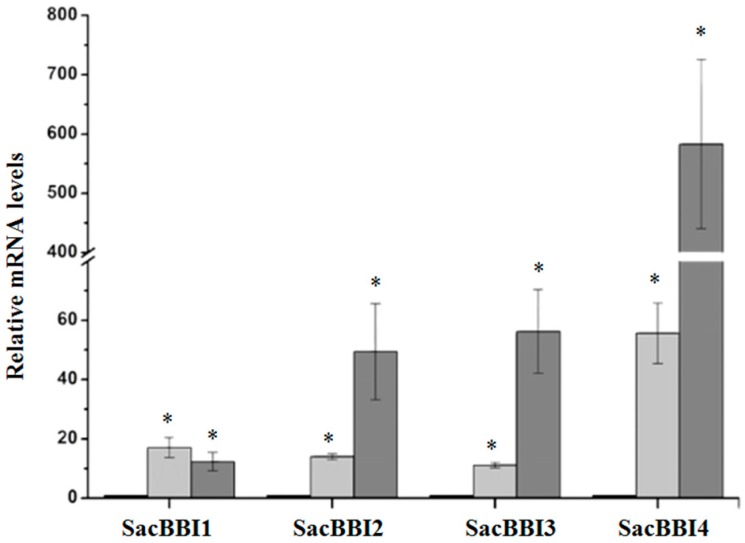
The relative expression levels of sugarcane Bowman-Birk peptidase inhibitors after 9 h of *D. saccharalis* feeding (dark gray bars), mechanical damage (light-gray bars), and control treatment (black bars). The expression levels were quantified by real-time quantitative PCR. The *x*-axis indicates the four sugarcane BBIs. The *y*-axis indicates the fold change in gene expression. The values are the means (±standard errors) of the transcripts from three replications, normalized to the transcript abundance of GAPDH. The regulation of expression was calculated using REST 2008 software [56]. The asterisks above the bars represent significant differences compared with the control at 0 h at a significance level of α < 0.05.

Four *D. saccharalis*-inducible sugarcane genes are homologous to rice and maize Bowman-Birk peptidase inhibitors and are designated here as SacBBI1-4 (Table 1). Real-time quantitative PCR analysis of the SacBBI genes showed that all four SacBBIs are induced by *D. saccharalis* and mechanical wounding (Figure 1). SacBBI1 is more responsive to mechanical wounding (17 times more highly expressed than the control) than caterpillar feeding (12 times more highly expressed than the control). SacBBI4 was induced by both caterpillar feeding and mechanical wounding; however, in contrast to SacBBI1, SacBBI4 mRNA levels in plants subjected to *D. saccharalis* feeding were approximately 600 times higher than the levels observed in control undisturbed plants and more than 50 times higher when plants were mechanically wounded (Figure 1). The other two genes, SacBBI2 and SacBBI3, were also induced by wounding and insect feeding, but to a lesser extent. Both the SacBBI2 and SacBBI3 genes were approximately 50 times more highly expressed in plants attacked by the insect and approximately 12 times more highly expressed in plants subjected to wounding.

We further investigated the behavior of all sugarcane BBIs present on the array, independent of their macroarray expression pattern. Gene expression quantification of 14 sugarcane BBI homologs confirmed that the four BBIs identified by our macroarray (SacBBI1 to 4) were the only BBIs induced by mechanical wounding or *D. saccharalis* feeding. The expression of the remaining 10 genes was unaltered, regardless of the treatment (data not shown).

#### 2.2.2. Sugarcane Maize-peptidase-inhibitor-like (SacMPI-like) and Chymotrypsin Inhibitor 1B-like (SacCI1B-like) Genes

**Figure 2:**
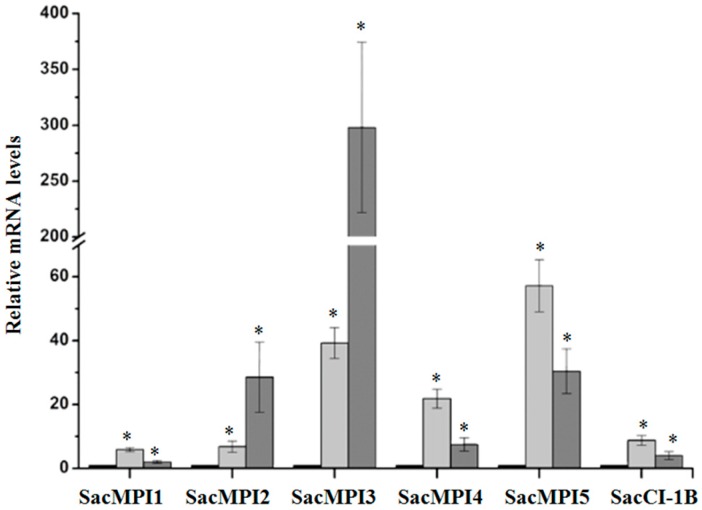
The relative expression levels of sugarcane maize-peptidase-inhibitor-like genes and the chymotrypsin inhibitor 1B-like gene after 9 h of *D. saccharalis* feeding (dark gray bars), mechanical damage (light-gray bars), and control treatment (black bars). The expression levels were quantified by real-time quantitative PCR. The *x*-axis indicates the six sugarcane serine peptidase inhibitors. The *y*-axis indicates the fold change in gene expression. The values are the means (±standard errors) of the transcripts from three replications, normalized to the transcript abundance of GAPDH. The regulation of expression was calculated using REST 2008 software [56]. The asterisks above the bars represent significant differences compared with the control at 0 (zero) h at a significance level of α < 0.05.

Changes in the mRNA levels of five sugarcane maize-peptidase-inhibitor-like genes (SacMPI-like1-5) and one chymotrypsin inhibitor 1B-like gene (SacCI1B-like) in response to *D. saccharalis* attack and mechanical wounding were evaluated. All SacMPI-like and SacCI1B-like genes were responsive to insect attack and wounding (Figure 2). Insect attack induced higher levels of SacMPI-like2 gene expression (28 times more highly expressed than in the control plants) than did wounding (seven times more highly expressed than in control plants). SacMPI-like 3 exhibited the highest gene induction by *D. saccharalis* feeding, with mRNA levels averaging 300 times greater than control levels when plants were damaged by *D. saccharalis* and approximately 60 times greater when plants were mechanically damaged. SacMPI-like1, 4, 5, and SacC1B-like were more responsive to mechanical wounding than insect attack.

All SacMPI-like genes are homologs of the I13 peptidase inhibitor family. To test the possible relationship between SacMPI-like gene evolution and its expression profile, we further investigated the distribution of all sugarcane SacMPI-like sequences within the I13 family via phylogenetic analysis (Supplementary Materials, Figure S2). I13 peptidase inhibitor homologs from sugarcane did not group based on their expression profiles (Supplementary Materials, Figure S2).

#### 2.2.3. Serine Peptidases and Clp Protease System Subunits

**Figure 3:**
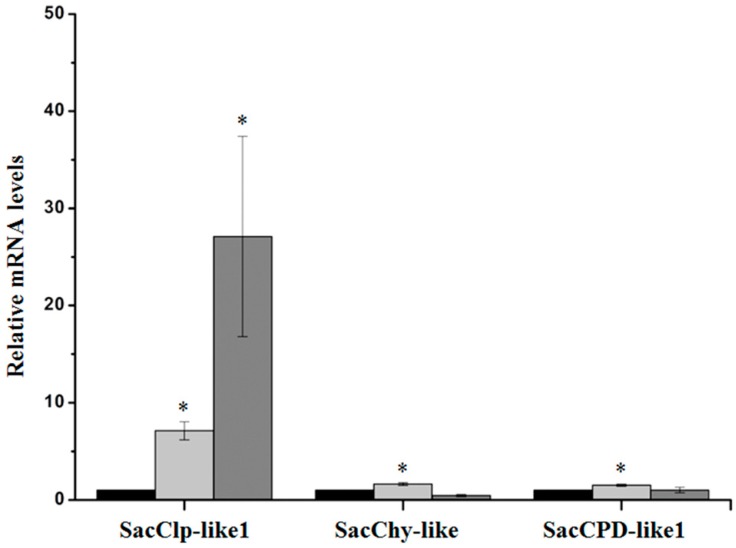
The relative expression levels of one sugarcane Clp subunit and two serine peptidases after 9 h of *D. saccharalis* feeding (dark gray bars), mechanical damage (light-gray bars), and control treatment (black bars). The expression levels were quantified by real-time quantitative PCR. The *x*-axis indicates the three sugarcane genes. The *y*-axis indicates the fold change in gene expression. The values are the means (±standard errors) of the transcripts from three replications, normalized to the transcript abundance of GAPDH. The regulation of expression was calculated using REST 2008 software [56]. The asterisks above the bars represent significant differences compared with the control at 0 (zero) h at α < 0.05 significance level.

Only two sugarcane serine peptidases and one Clp subunit were identified as induced by *D. saccharalis* in our macroarray, and their induction by herbivory was validated by performing quantitative real-time PCR analysis (Figure 3).

Among the 16 sugarcane Clp-like subunits analyzed in the macroarray, only the SacClp-like1 subunit (homolog of the *Arabidopsis* ClpD AAA+ chaperone subunit) was significantly induced by both wounding and insect attack (*p* < 0.05) (Table 3). Both SacChy-like and SacCPD-like1 were marginally induced by mechanical wounding. The insect wounding was not statistically different from control plants (at level of *p* < 0.05). SacClp-like1 gene was about 30 times more expressed in *D. saccharalis*-wounded leaves than in leaves of undisturbed control plants (Figure 3). Mechanical damage caused an increase of the SacClp-like1 mRNA levels up to seven times the levels found in control plants.

### 2.3. Validation of D. saccharalis-Repressed Genes

Nine sugarcane genes were repressed by *D. saccharalis* feeding, with the highest reduction observed for the homolog of the *Zea mays* serine carboxypeptidase K10B2.2 [57] (SacCPD-like3) (Table 4). The sugarcane homolog of the *Arabidopsis* ClpC2 chaperone subunit of the Clp protease system (SacClp-like3) exhibited a 6.4-fold reduction in mRNA levels in *D. saccharalis*-treated plants. A sugarcane homolog of a putative subtilase family protein of *Zea mays* [58] (SacSub-like1) demonstrated a 4.4-fold reduction in insect-attacked plants. The remaining six genes exhibited repression levels below 3-fold.

## 3. Discussion

In this work, a customized macroarray containing 248 selected sugarcane genes putatively encoding serine peptidase inhibitors, serine peptidases, and Clp protease system subunits was used to identify sugarcane genes involved in the response to *D. saccharalis* herbivory. The macroarray technique used in this work has proven to be an elegant, rapid, and low-cost method to obtain the sugarcane transcript profile following *D. saccharalis* feeding. We identified 13 up- and 9 downregulated sugarcane homologs of serine peptidases and serine peptidase inhibitors (Table 1 and Table 2).

Wounding of plant leaves, either by insects or mechanical injury, induces the rapid accumulation of peptidase inhibitors throughout the plant, in both damaged and adjacent tissues [10,49,59,60]. We hypothesized that when *D. saccharalis* feeds on sugarcane, it will trigger the accumulation of sugarcane serine peptidase inhibitors because *D. saccharalis* possesses an alkaline pH in its mesenterium, where serine peptidases are most active [20].

Our macroarray results showed that four sugarcane genes homologous to BBIs (SacBBI1 to 4) were induced in response to sugarcane borer herbivory (Figure 1 and Table 1). qPCR positively validated the expression of these genes and revealed that the increases in their expression ranged from 12 to 582 times the levels detected in undisturbed plants. The SacBBI2 gene was previously reported to be induced by insect and mechanical wounding [49]. The remaining 10 sugarcane BBI genes represented in the macroarray were not induced by *D. saccharalis* feeding. Studies examining the molecular evolution of this group have shown that the sugarcane BBIs can be divided into six subgroups based on amino acid sequence similarity [19]. Curiously, all four sugarcane BBIs induced by wounding diverge phylogenetically from the other ten BBIs that maintained constant expression. We speculate that our phylogenetic analysis, which grouped the BBI sequences into six groups, is accompanied by functional similarity. Some groups have diverged to fulfill a specific biological role in response to wounding (i.e., the four BBIs identified by our study), whereas other groups might possess other biological roles unrelated to plant defense (i.e., the 10 BBIs present in the macroarray that were not induced by wounding).

In addition to the four BBI genes that have already been discussed, our macroarray identified five genes with similarity to the potato inhibitor type I family, including the Maize Proteinase Inhibitor (MPI) and a gene similar to a subtilisin-chymotrypsin inhibitor (Figure 2 and Table 1). MPIs have been shown to contribute to plant insect defense and are induced by insect and mechanical wounding [52,59,60,61,62,63]. The gene SacMPI-like2 CI-1 exhibits strong similarity to the subtilisin-chymotrypsin inhibitor CI-1B. In barley, the related chymotrypsin inhibitor family (CI-2) is associated with pathogen defense [64,65]. The inhibitors identified here may be candidates for incorporation into plant biotechnology programs. For example, when feeding on transgenic sugarcane overexpressing BBI and Kunitz-type PIs, *D. saccharalis* suffers diminished growth and metabolism [66]. In addition, *D. saccharalis* larvae raised on an artificial diet supplemented with peptidase inhibitors exhibit diminished growth and development and low fecundity rates [67,68].

Although serine peptidases and Clp subunits represented 81% of the genes present in our array, only two serine peptidases and only one Clp subunit were induced by *D. saccharalis* feeding (Figure 3 and Table 1).

Our results show that one putative sugarcane Clp protease system subunit (SacClp-like) was highly induced by herbivory. This subunit is homologous to the *Arabidopsis* Clp AAA+ chaperone subunit ClpD. Interestingly, three other sugarcane homologs of *Arabidopsis* Clp subunits were downregulated. The Clp protease system plays an important role in chloroplast protein homeostasis and metabolism [69], and *Arabidopsis* Clp subunits were previously reported to be involved in responses to light and cold acclimation [70,71].

The genes identified in this work can be further characterized and potentially used as molecular markers in sugarcane breeding programs [46] or as candidate genes for transgenic approaches for sugarcane improvement [40], which has been carried out for apples, tomatoes, and previously in sugarcane [66,72,73].

## 4. Materials and Methods

### 4.1. Plant Material and Insects

#### 4.1.1. Phase 1—Sugarcane and *D. saccharalis* Experiments Used for Macroarray Hibridization

Sugarcane plants (*Saccharum* hybrid cultivar SP80-3280) were kindly provided by Centro de Tecnologia Canavieira (CTC), Piracicaba, SP, Brazil. Sugarcane plants were obtained from vegetative stalk cuttings called setts (nodal buds). One-eyed setts were planted in 200-mL plastic cups containing a commercial planting mix (Plantmax, Eucatex) and cultivated in a greenhouse at temperatures ranging from 18 (night) to 34 °C (day). *Diatraea saccharalis* was kindly provided by Centro de Tecnologia Canavieira, Piracicaba, SP, Brazil. The caterpillars were maintained on an artificial diet at 25 °C, 60% ± 10% relative humidity, and a 14-h photophase.

Biological experiments were conducted in the greenhouse facility of the Department of Genetics of the ESALQ (Escola Superior de Agricultura “Luiz de Queiroz”). To monitor sugarcane SP80-3280 transcript profiles after *D. saccharalis* attack, three treatments were applied: (A) non-attacked plants at the 0 h (time point); (B) non-attacked plants at the 9 h time point; and (C) *D. saccharalis*-attacked plants at the 9 h time point. Fourth-instar caterpillars were removed from diet and kept without food for 24 h before the beginning of the experiment. At this time, each caterpillar was carefully transferred with the aid of forceps, to the base of the stalk of each 20-day-old sugarcane plant. The feeding behavior of the caterpillar was checked for 20 minutes; if it failed to start feeding, the caterpillar was discarded and replaced. At the end of the experiment, the insects were removed. Each treatment had three biological replications, each consisting of a pool of four plants. After each time point, the entire aerial upper portion of the four plants of the replicate was collected, bulked, immediately frozen in liquid nitrogen, and RNA was extracted from this pool of plants. The entire biological experiment was repeated twice to confirm the first result.

#### 4.1.2. Phase 2—qPCR Monitoring of Selected ESTs Identified through Macroarray

Expression of sugarcane ESTs identified through macroarray was validated through real-time qPCR. To perform this, another set of independent biological experiments was conducted.

The experiment was performed as described before (in phase 1), with few modifications. A mechanically-injured treatment was added. Thirty-day-old sugarcane plants were used, and the treatments were: (A) non-attacked plants at the 0 time point; (B) insect attacked plants at the 9 h time point; and (C) mechanically-injured plants at the 9 h time point. In the mechanical injury group, the plants were wounded repeatedly every hour with fine forceps, for 9 h.

### 4.2. Macroarray Construction

Our macroarray was built by the Brazilian Clone Collection Center using the sugarcane genes of 248 peptidase inhibitors, serine peptidases, and Clp protease system subunits selected from the SUCEST (Sugarcane EST Sequencing Project) database. The serine peptidases and Clp subunits comprised 81% of the total genes, whereas the peptidase inhibitors comprised 13% of the genes. The other 6% were represented by reference genes such as β-actin, GAPDH, eukaryotic initiation factor (eIF) and ubiquitin. The complete list of genes spotted onto the array is given in Supplemental Table S1.

The array was spotted onto six nitrocellulose membranes. The spot pattern consisted of a 3 × 3 array, and each sub-array contained two genes and an empty central spot. Each gene was spotted in quadruplicate. The macroarray technique used was established for filter-based methods [74].

### 4.3. Macroarray Normalization

To normalize the amount of DNA spotted on the filter membrane and to monitor the amount of DNA that was washed away after probe stripping as well as before and after cDNA probe hybridization, the macroarrays were hybridized with a probe designed to hybridize with a common region of the plasmid vector, specifically the Amp^r^ gene sequence [75] of the pSPORT1 vector, that is used to build the SUCEST libraries [41].

Probes were synthesized with the primers 5′-GTGGTCCTGCAACTTTATCCGC-3′ and 5′-TAGACTGGATGGAGGCGGATAA-3′ in the presence of [α-^33^P] dCTP for 1 h at room temperature. After purification using ProbeQuant G-50 microcolumns according to the manufacturer’s instructions (Amersham Biosciences, St. Catherine, ON, Canada), the probe was denatured for 3 min at 94 °C and added to the hybridization solution. All filters were placed in the same container, and pre-hybridization was performed for 4 h at 58 °C, followed by 18 h of hybridization (200 mL of 1% BSA, 0.5 mM EDTA (pH 8), 7% SDS, 1 M sodium phosphate (pH 7.2)). The filters were washed with decreasing concentrations of SSC. After washing, filter membranes were sealed with plastic film and were immediately exposed to imaging plates (Fuji Photo Film Co., Tóquio, Japan) for 72 h. Intensity signals were captured using a Storm 860 PhosphorImager (Bio-Rad, Berkeley, CA, USA). Next, the oligo vector probes were removed from the filters as described in [76]. The efficiency of probe removal was monitored by phosphorimager scanning after membrane filter exposition to imaging plates (Fuji Photo Film Co., Tóquio, Japan) for 72 h.

The median values of the signal intensities for all spots were determined. The variation coefficient of those values was estimated to assess the amount of DNA fluctuation among replicates [77]. Only those replicates with variation coefficients lower than 10% were used for subsequent analysis.

### 4.4. Probe Preparation

The filter membranes were probed with ^33^P cDNA populations derived from RNA extracted from the leaves of undisturbed sugarcane plants (0 and 9 h time points) and plants attacked by *D. saccharalis* (9 h time point). To reduce variation among replicate filters, each of the three probes was hybridized, one at a time, with each of the three filter membranes over three rounds of hybridization. To do so, we produced the three probes once, divided the preparations into three aliquots and kept them frozen until hybridization was carried out. After hybridization and the measurement of hybridization signals, only those genes that presented the same expression pattern (up- or downregulation) in at least two hybridizations among the six possible comparisons were selected.

To prepare the probes, total RNA from above-ground sugarcane tissue was used. RNA was extracted using TRIZOL^®^ reagent (Invitrogen, Waltham, MA, USA), followed by deoxyribonucleic acid removal with two units of RNAse-free DNase I (Fermentas, Waltham, MA. USA) at 37 °C for 20 min. The RNA was re-extracted and then quantified by spectrophotometer. The RNA quality was checked by gel electrophoresis. Probes were produced by the reverse transcription of 30 μg of total RNA using SuperScript III (Invitrogen, Waltham, MA, USA) as well as 50 μCi of alphaP^33^-dCTP and unlabeled dATP, dGTP, and dTTP, following the protocol of Schummer et al. [76]. The cDNA probes were purified by using ProbeQuant G-50 microcolumns according to the manufacturer’s instructions (Amersham Biosciences, St. Catherine, ON, Canada). The probes were synthesized, purified, divided into three fractions, and frozen at −20 °C. For each cycle of hybridization, a new fraction was defrosted for use. Pre-hybridization, hybridization, and washing were performed as described in the Southern protocol [78]. After washing, filter membranes were sealed with plastic film and were immediately exposed to imaging plates (Fuji Photo Film Co., Tóquio, Japan) for 72 h. The intensity signals were captured using a Storm 860 PhosphorImager (Bio-Rad, Berkeley, CA, USA). Then, stripping was performed as described by Schummer et al. [76].

### 4.5. Macroarray Analysis

All signals were quantified using ArrayVision 8.0 rev 5.0 software (Imaging Research, London, ON, Canada). The grids were predefined and adjusted to obtain optimal spot recognition. For each spot, the AR volume (the spot density, minus artifacts and multiplied by its area), background (background pixel median density), and nARVOL (normalized AR volume) were measured. ArrayVision files were exported and opened using PMmA software [77]. Array normalization was performed with the Arrayflags script using the median of the overgo probe data. Only those genes for which the intensity signal average did not vary significantly among the four replicates were further used. Data analysis was performed using the algorithm ISER [79], which calculates and normalizes the signal intensity geometric average and ratio between the treatment and control. Genes were considered upregulated if the ratio was above the upper limit of the signal intensity threshold. Genes were considered downregulated if the ratio between the treatment and control signals was below the lower limit of the threshold.

### 4.6. Expression Analysis by Real-Time Quantitative PCR

Total RNA of each replicate was extracted with TRIZOL as described earlier in the macroarray section.

First strand synthesis was performed using Improm-II™ Reverse Transcriptase (Promega, Madison, WI, USA), according to the manufacturer recommendations, from 2 µg of total RNA, in a total volume of 20 µL. Real-time quantitative PCR was performed in a StepOne™ equipment (Applied Biosystems, Waltham, MA, USA) with Platinum SYBR Green qPCR SuperMix-UDG (Invitrogen, Waltham, MA, USA) reagent, following the recommendations of the manufacturer. The primer annealing temperature used was 62 °C and the fluorescence signal was captured at the end of each cycle. The melting curve analysis was performed from 72 to 99 °C, holding for 45 s during the first step and holding for 5 s during subsequent steps.

Primers for qPCR were designed based on the up- and downregulated genes. The Supplemental Table S2 presents the primer sequences used in this analysis. Data analysis was performed using the Pfaffl method [80]. The threshold was manually defined as 0.1 of the normalized fluorescence. Statistical analyses were performed using the Pair Wise Fixed Reallocation Randomization Test © [80] using 2000 randomizations and adopting *p* < 0.05 as the significance value.

## Figures and Tables

**Table 1:** Genes upregulated after 9 h of *D. saccharalis* feeding.

Sugarcane Clone Identification ^a^	*E*-Value	Identity (%)	BLAST Hit ^b^	Description ^c^
SacBBI1 GI: 34966865 GB: CA113558.1	2 × 10^−24^	73/87 (84%)	GI:195610004	Bowman–Birk type wound-induced proteinase inhibitor WIP1 precursor [*Zea mays*]
SacBBI2 GI: 35951517 GB: CA261007.1	1 × 10^−40^	85/98 (87%)	GI:115434342	Bowman–Birk type proteinase inhibitor *Oryza sativa Japonica* Group
SacBBI3 GI: 35965021 GB: CA266304.1	5 × 10^−43^	74/88 (84%)	GI:195610814	Bowman–Birk type bran trypsin inhibitor precursor [*Zea mays*]
SacBBI4 GI: 35984624 GB: CA272687.1	2 × 10^−39^	85/98 (87%)	GI:115434342	Bowman–Birk type proteinase inhibitor *Oryza sativa Japonica* Group
SacMPI-like1 GI: 34922345 GB: CA070500.1	4 × 10^−26^	51/60 (85%)	GI:214015177	Maize proteinase inhibitor [*Zea mays* subsp. *parviglumis*]
SacMPI-like2 GI: 35258606 GB: CA212876.1	6 × 10^−33^	59/66 (89%)	GI:75994161	Maize protease inhibitor [*Zea mays* subsp. *parviglumis*]
SacMPIlike3 GI: 36014330 GB:CA282462.1	1 × 10^−31^	58/70 (83%)	GI:214015219	Maize proteinase inhibitor [*Zea mays* subsp. *parviglumis*]
SacMPI-like4 GI: 36037506 GB: CA288211.1	9 × 10^−29^	55/65 (85%)	GI:214015093	Maize proteinase inhibitor [*Zea mays* subsp. *parviglumis*]
SacMPI-like5 GI: 36065043 GB:CA297188.1	2 × 10^−28^	56/65 (85%)	GI:214015177	Maize proteinase inhibitor [*Zea mays* subsp. *parviglumis*]
SacCI-1B-like GI: 35010896 GB: CA129230.1	0.067	17/26 (65%)	GI:226507138	Subtilisin-chymotrypsin inhibitor CI-1B [*Zea mays*]
SacClp-like1 CA136349.1	2 × 10^−77^	94/94 (100%)	GI:242061800	Clp amino terminal domain; *Sorghum bicolor*
SacChy-like GI: 35946811 GB: CA258670.1	1 × 10^−153^	215/221 (97%)	GI:242077536	PDZ domain of trypsin-like serine proteases
SacCPD-like1 GI: 36002999 GB: CA278685.1				No significant similarity found

^a^ GI: Gene Identification number and GB: GenBank Accession number; ^b^ BLAST hit was obtained using the BLASTX algorithm [51]; ^c^ Description indicates the putative functions of gene products expected from similar sequences.

**Table 2:** Genes downregulated after 9 h of *D. saccharalis* feeding.

Sugarcane Clone Identification ^a^	*E*-Value	Identity (%)	BLAST Hit ^b^	Description ^c^
SacClp-like2 GI: 34940929 GB: CA087622.1	1 × 10^−137^	192/198 (97%)	GI:195612324	ATP-dependent Clp protease proteolytic subunit 2 [*Zea mays*]
SacClp-like3 GI: 34966311 GB: CA113004.1	4 × 10^−143^	208/224 (93%)	GI:413935895	Putative chaperone clp family protein [*Zea mays*]
SacClp-like4 GI: 35005555 GB: CA126553.1	5 × 10^−62^	131/150 (87%)	GI:347602486	ATP-dependent Clp protease ATP-binding subunit ClpC homolog 1, *Oryza sativa Japonica* Group
SacClp-like5 GI: 35081269 GB: CA164148.1	7 × 10^−108^	180/189 (95%)	GI:475585607	ATP-dependent Clp protease ATP-binding subunit clpA-like CD4A protein, chloroplastic [*Aegilops tauschii*]
SacCPD-like2 GI: 34966324 GB: CA113017.1	4 × 10^−127^	177/198 (89%)	GI:195637388	Serine carboxypeptidase K10B2.2 precursor [*Zea mays*]
SacCPD-like3 GI: 35050806 GB: CA149102.1	7 × 10^−58^	94/105 (90%)	GI:226507958	Serine carboxypeptidase K10B2.2 precursor [*Zea mays*]
SacSub-like1 GI: 34948297 GB: CA094990.1	2 × 10^−136^	199/221 (90%)	GI:414880317	TPA: putative subtilase family protein [*Zea mays*]
SacSub-like2 GI: 34967468 GB: CA114161.1	2 × 10^−42^	80/100 (80%)	GI:42407651	Putative subtilisin-like proteinase [*Oryza sativa Japonica* Group]
SacSub-like3 GI: 34967945 GB: CA114638.1	6 × 10^−76^	124/158 (78%)	GI:475577050	Subtilisin-like protease [*Aegilops tauschii*]

^a^ GI: Gene Identification number and GB: GenBank accession number; ^b^ BLAST hit was obtained using the BLASTX algorithm [51]; ^c^ Description indicated the putative functions of gene products expected from similar sequences.

**Table 3:** Expression profiles of sugarcane Clp protease system subunit homologs after herbivore attack.

Role	*Arabidopsis* Subunit	Accession	Sugarcane EST Homologue	Expression Profile *
Clp AAA^+^ chaperones	ClpC1	AT5G50920	CA119085.1	-
			CA124181.1	-
			CA126553.1 (SacClp-like4)	−1.9 ± 0.07
			CA132637.1	-
			CA164148.1 (SacClp-like5)	−1.9 ± 0.06
	ClpC2	AT3G48870	CA113004.1 (SacClp-like3)	−6.4 ± 0.03
	ClpD	AT5G51070	CA119462.1	-
			CA136349.1 (SacClp-like1)	27,11 ± 10,31
			CA145821.1	-
			CA194919.1	-
			CA212375.1	-
serine-type ClpP	ClpP1	ATCG00670	CA119497.1	-
	ClpP3	AT1G66670	CA119729.1	-
	ClpP4	AT5G45390	CA107353.1	-
	ClpP5	AT1G02560	CA074329.1	-
			CA183086.1	-
	ClpP6	AT1G11750	CA282400.1	-
	ClpP7	AT5G23140	-	-
ClpP-related ClpR	ClpR1	AT1G49970	CA108609.1	-
			CA108695.1	-
	ClpR2	AT1G12410	CA087622.1 (SacClp-like2)	−2.1 ± 0.14
	ClpR3	AT1G09130	Not found	
	ClpR4	AT4G17040	CA113615.1	-

* *p*-value < 0.005.

**Table 4:** Relative levels of gene repression after 9 h of *D. saccharalis* feeding.

Sugarcane Clone Name	GenBank Accession Number	Relative mRNA Level	*p* Value
SacClp-like2	CA087622.1	‒2.1 ± 0.14	*p* < 0.005
SacClp-like3	CA113004.1	‒6.4 ± 0.03	*p* < 0.005
SacClp-like4	CA126553.1	‒1.9 ± 0.07	*p* < 0.005
SacClp-like5	CA164148.1	‒1.9 ± 0.06	*p* < 0.005
SacCPD-like2	CA113017.1	‒2.1 ± 0.07	*p* < 0.005
SacCPD-like3	CA149102.1	‒44.2 ± 0.008	*p* < 0.005
SacSub-like1	CA094990.1	‒4.4 ± 0.04	*p* < 0.005
SacSub-like2	CA114161.1	‒2.7 ± 0.08	*p* < 0.005
SacSub-like3	CA114638.1	‒1.6 ± 0.06	*p* < 0.005

## Supplementary Materials

Supplementary materials can be found at www.mdpi.com/1422-0067/17/9/1444/s1.

## References

[1] Vendramim J., da Silva F., César M., de Camargo A. (1988). Comparação entre dois métodos para avaliação da infestação pelo complexo broca-podridões em cultivares de cana-de-açúcar. Anais da Escola Superior de Agricultura Luiz de Queiroz.

[2] Ogunwolu E.O., Reagan T.E., Flynn J.L., Hensley S.D. (1991). Effects of *Diatraea saccharalis* (F.) (*Lepidoptera*: *Pyralidae*) damage and stalk rot fungi on sugarcane yield in louisiana. Crop Prot..

[3] Schmelz E.A., Carroll M.J., LeClere S., Phipps S.M., Meredith J., Chourey P.S., Alborn H.T., Teal P.E.A. (2006). Fragments of ATP synthase mediate plant perception of insect attack. Proc. Natl. Acad. Sci. USA.

[4] Mattiacci L., Dicke M., Posthumus M.A. (1995). Beta-glucosidase: An elicitor of herbivore-induced plant odor that attracts host-searching parasitic wasps. Proc. Natl. Acad. Sci. USA.

[5] Turlings T.C.J., Tumlinson J.H., Lewis W.J. (1990). Exploitation of herbivore-induced plant odors by host-seeking parasitic wasps. Science.

[6] Dangl J.L., Jones J.D.G. (2001). Plant pathogens and integrated defence responses to infection. Nature.

[7] Smith C.M., Boyko E.V. (2007). The molecular bases of plant resistance and defense responses to aphid feeding: Current status. Entomol. Exp. Appl..

[8] Mithofer A., Boland W. (2008). Recognition of herbivory-associated molecular patterns. Plant Physiol..

[9] Felton G.W., Tumlinson J.H. (2008). Plant-insect dialogs: Complex interactions at the plant-insect interface. Curr. Opin. Plant Biol..

[10] Green T.R., Ryan C.A. (1972). Wound-induced proteinase inhibitor in plant leaves: A possible defense mechanism against insects. Science.

[11] Hartl M., Giri A.P., Kaur H., Baldwin I.T. (2011). The multiple functions of plant serine protease inhibitors: Defense against herbivores and beyond. Plant Signal. Behav..

[12] Jongsma M.A., Beekwilder J. (2011). Co-evolution of insect proteases and plant protease inhibitors. Curr. Protein Pept. Sci..

[13] Ryan C.A. (1990). Protease inhibitors in plants: Genes for improving defenses against insects and pathogens. Annu. Rev. Phytopathol..

[14] Bowman D.E. (1946). Differentiation of soy bean antitryptic factors. Proc. Soc. Exp. Biol. Med..

[15] Birk Y., Gertler A., Khalef S. (1963). A pure trypsin inhibitor from soya beans. Biochem. J..

[16] Norioka S., Ikenaka T. (1983). Amino acid sequence of a trypsin-chymotrypsin inhibitor, B-III, of peanut (*Arachis Hypogaea*). J. Biochem..

[17] Tanaka A.S., Sampaio M.U., Marangoni S., deOliveira B., Novello J.C., Oliva M.L.V., Fink E., Sampaio C.A.M. (1997). Purification and primary structure determination of a bowman-birk trypsin inhibitor from *Torresea Cearensis* seeds. Biol. Chem..

[18] Odani S., Koide T., Teruo O. (1986). Wheat germ trypsin inhibitors. Isolation and structural characterization of single-headed and double-headed inhibitors of the bowman-birk type. J. Biochem..

[19] Mello M.O., Tanaka A.S., Silva-Filho M.C. (2003). Molecular evolution of bowman-birk type proteinase inhibitors in flowering plants. Mol. Phylogenet. Evol..

[20] Terra W.R., Ferreira C. (1994). Insect digestive enzymes—Properties, compartmentalization and function. Comp. Biochem. Physiol. Part B.

[21] Botella M.A., Xu Y., Prabha T.N., Zhao Y., Narasimhan M.L., Wilson K.A., Nielsen S.S., Bressan R.A., Hasegawa P.M. (1996). Differential expression of soybean cysteine proteinase inhibitor genes during development and in response to wounding and methyl jasmonate. Plant Physiol..

[22] Reymond P., Weber H., Damond M., Farmer E.E. (2000). Differential gene expression in response to mechanical wounding and insect feeding in arabidopsis. Plant Cell.

[23] Little D., Gouhier-Darimont C., Bruessow F., Reymond P. (2007). Oviposition by pierid butterflies triggers defense responses in arabidopsis. Plant Physiol..

[24] Van der Hoorn R.A.L., Jones J.D. (2004). The plant proteolytic machinery and its role in defence. Curr. Opin. Plant Biol..

[25] Moura D.S., Bergey D.R., Ryan C.A. (2001). Characterization and localization of a wound-inducible type i serine-carboxypeptidase from leaves of tomato plants (*Lycopersicon esculentum* Mill.). Planta.

[26] Tornero P., Conejero V., Vera P. (1996). Primary structure and expression of a pathogen-induced protease (PR-P69) in tomato plants: Similarity of functional domains to subtilisin-like endoproteases. Proc. Natl. Acad. Sci. USA.

[27] Horn M., Patankar A.G., Zavala J.A., Wu J.Q., Doleckova-Maresova L., Vujtechova M., Mares M., Baldwin I.T. (2005). Differential elicitation of two processing proteases controls the processing pattern of the trypsin proteinase inhibitor precursor in nicotiana attenuata. Plant Physiol..

[28] Schaller A., Ryan C.A. (1994). Identification of a 50-kDa systemin-binding protein in tomato plasma-membranes having Kex2p-like properties. Proc. Natl. Acad. Sci. USA.

[29] Pearce G., Strydom D., Johnson S., Ryan C.A. (1991). A polypeptide from tomato leaves induces wound-inducible proteinase-inhibitor proteins. Science.

[30] Ryan C.A., Moura D.S. (2002). Systemic wound signaling in plants: A new perception. Proc. Natl. Acad. Sci. USA.

[31] Pechan T., Cohen A., Williams W.P., Luthe D.S. (2002). Insect feeding mobilizes a unique plant defense protease that disrupts the peritrophic matrix of caterpillars. Proc. Natl. Acad. Sci. USA.

[32] Pechan T., Ye L., Chang Y.-m., Mitra A., Lin L., Davis F.M., Williams W.P., Luthe D.S. (2000). A unique 33-kD cysteine proteinase accumulates in response to larval feeding in maize genotypes resistant to fall armyworm and other lepidoptera. Plant Cell.

[33] Mohan S., Ma P.W.K., Pechan T., Bassford E.R., Williams W.P., Luthe D.S. (2006). Degradation of the *S. frugiperda* peritrophic matrix by an inducible maize cysteine protease. J. Insect. Physiol..

[34] Pautot V., Holzer F.M., Chaufaux J., Walling L.L. (2001). The induction of tomato leucine aminopeptidase genes (*LapA*) after *Pseudomonas syringae* pv. *tomato* infection is primarily a wound response triggered by coronatine. Mol. Plant Microbe Interact..

[35] Chao W.S., Gu Y.Q., Pautot V., Bray E.A., Walling L.L. (1999). Leucine aminopeptidase rnas, proteins, and activities increase in response to water deficit, salinity, and the wound signals systemin, methyl jasmonate, and abscisic acid. Plant Physiol..

[36] Fowler J.H., Narváez-Vásquez J., Aromdee D.N., Pautot V., Holzer F.M., Walling L.L. (2009). Leucine aminopeptidase regulates defense and wound signaling in tomato downstream of jasmonic acid. Plant Cell.

[37] Chen H., Wilkerson C.G., Kuchar J.A., Phinney B.S., Howe G.A. (2005). Jasmonate-inducible plant enzymes degrade essential amino acids in the herbivore midgut. Proc. Natl. Acad. Sci. USA.

[38] Gonzales-Vigil E., Bianchetti C.M., Phillips G.N., Howe G.A. (2011). Adaptive evolution of threonine deaminase in plant defense against insect herbivores. Proc. Natl. Acad. Sci. USA.

[39] Kessler A., Baldwin I.T. (2002). Plant responses to insect herbivory: The emerging molecular analysis. Annu. Rev. Plant Biol..

[40] Chandra A., Jain R., Solomon S., Shrivastava S., Roy A.K. (2013). Exploiting EST databases for the development and characterisation of 3425 gene-tagged CISP markers in biofuel crop sugarcane and their transferability in cereals and orphan tropical grasses. BMC Res. Notes.

[41] Vettore A.L., da Silva F.R., Kemper E.L., Arruda P. (2001). The libraries that made sucest. Genet. Mol. Biol..

[42] Vettore A.L., da Silva F.R., Kemper E.L., Souza G.M., da Silva A.M., Ferro M.I.T., Henrique-Silva F., Giglioti E.A., Lemos M.V.F., Coutinho L.L. (2003). Analysis and functional annotation of an expressed sequence tag collection for tropical crop sugarcane. Genome Res..

[43] Casu R., Dimmock C., Thomas M., Bower N., Knight D., Grof C., McIntyre L., Jackson P., Jordan D., Whan V. (2001). Genetic and expression profiling in sugarcane. Proc. Int. Soc. Sugar Cane Technol..

[44] Carson D.L., Huckett B.I., Botha F.C. (2002). Sugarcane ests differentially expressed in immature and maturing internodal tissue. Plant Sci..

[45] Ma H.M., Schulze S., Lee S., Yang M., Mirkov E., Irvine J., Moore P., Paterson A. (2004). An est survey of the sugarcane transcriptome. Theor. Appl. Genet..

[46] Menossi M., Silva-Filho M.C., Vincentz M., Van-Sluys M.A., Souza G.M. (2008). Sugarcane functional genomics: Gene discovery for agronomic trait development. Int. J. Plant Genom..

[47] Souza G.M., Simoes A.C.Q., Oliveira K.C., Garay H.M., Fiorini L.C., Gomes F.D., Nishiyama-Junior M.Y., da Silva A.M. (2001). The sugarcane signal transduction (SUCAST) catalogue: Prospecting signal transduction in sugarcane. Genet. Mol. Biol..

[48] Falco M.C., Marbach P.A.S., Pompermayer P., Lopes F.C.C., Silva-Filho M.C. (2001). Mechanisms of sugarcane response to herbivory. Genet. Mol. Biol..

[49] Medeiros A.H., Franco F.P., Matos J.L., de Castro P.A., Santos-Silva L.K., Henrique-Silva F., Goldman G.H., Moura D.S., Silva-Filho M.C. (2012). Sugarwin: A sugarcane insect-induced gene with antipathogenic activity. Mol. Plant Microbe Interact..

[50] Rocha F.R., Papini-Terzi F.S., Nishiyama M.Y., Vencio R.Z.N., Vicentini R., Duarte R.D.C., de Rosa V.E., Vinagre F., Barsalobres C., Medeiros A.H. (2007). Signal transduction-related responses to phytohormones and environmental challenges in sugarcane. BMC Genom..

[51] Altschul S.F., Gish W., Miller W., Myers E.W., Lipman D.J. (1990). Basic local alignment search tool. J. Mol. Biol..

[52] Lee J.S., Brown W.E., Graham J.S., Pearce G., Fox E.A., Dreher T.W., Ahern K.G., Pearson G.D., Ryan C. (1986). Molecular characterization and phylogenetic studies of a wound-inducible proteinase inhibitor i gene in lycopersicon species. Proc. Natl. Acad. Sci. USA.

[53] Bricchi I., Leitner M., Foti M., Mithofer A., Boland W., Maffei M.E. (2010). Robotic mechanical wounding (MecWorm) versus herbivore-induced responses: Early signaling and volatile emission in lima bean (*Phaseolus lunatus* L.). Planta.

[54] Howe G.A., Lightner J., Browse J., Ryan C.A. (1996). An octadecanoid pathway mutant (jl5) of tomato is compromised in signaling for defense against insect attack. Plant Cell.

[55] Alborn H.T., Turlings T.C.J., Jones T.H., Stenhagen G., Loughrin J.H., Tumlinson J.H. (1997). An elicitor of plant volatiles from beet armyworm oral secretion. Science.

[56] Pfaffl M.W., Horgan G.W., Dempfle L. (2002). Relative expression software tool (REST©) for group-wise comparison and statistical analysis of relative expression results in real-time PCR. Nucleic Acid Res..

[57] Alexandrov N.N., Brover V.V., Freidin S., Troukhan M.E., Tatarinova T.V., Zhang H., Swaller T.J., Lu Y.-P., Bouck J., Flavell R.B. (2009). Insights into corn genes derived from large-scale cdna sequencing. Plant Mol. Biol..

[58] Schnable P.S., Ware D., Fulton R.S., Stein J.C., Wei F., Pasternak S., Liang C., Zhang J., Fulton L., Graves T.A. (2009). The b73 maize genome: Complexity, diversity, and dynamics. Science.

[59] Cordero M.J., Raventos D., San Segundo B. (1994). Expression of a maize proteinase inhibitor gene is induced in response to wounding and fungal infection: Systemic wound-response of a monocot gene. Plant J..

[60] Tamayo M.C., Rufat M., Bravo J.M., San Segundo B. (2000). Accumulation of a maize proteinase inhibitor in response to wounding and insect feeding, and characterization of its activity toward digestive proteinases of spodoptera littoralis larvae. Planta.

[61] Plunkett G., Senear D.F., Zuroske G., Ryan C.A. (1982). Proteinase inhibitors I and II from leaves of wounded tomato plants: Purification and properties. Arch. Biochem. Biophys..

[62] Heath R.L., McDonald G., Christeller J.T., Lee M., Bateman K., West J., Van Heeswijck R., Anderson M.A. (1997). Proteinase inhibitors from *Nicotiana alata* enhance plant resistance to insect pests. J. Insect Physiol..

[63] Farag M.A., Fokar M., Abd H., Zhang H., Allen R.D., Pare P.W. (2005). (*Z*)-3-hexenol induces defense genes and downstream metabolites in maize. Planta.

[64] Wei F., Wing R.A., Wise R.P. (2002). Genome dynamics and evolution of the *Mla* (powdery mildew) resistance locus in barley. Plant Cell.

[65] Beßer K., Jarosch B., Langen G., Kogel K.H. (2000). Expression analysis of genes induced in barley after chemical activation reveals distinct disease resistance pathways. Mol. Plant Pathol..

[66] Falco M.C., Silva-Filho M.C. (2003). Expression of soybean proteinase inhibitors in transgenic sugarcane plants: Effects on natural defense against diatraea saccharalis. Plant Physiol. Biochem..

[67] Pompermayer P., Lopes A.R., Terra W.R., Parra J.R.P., Falco M.C., Silva-Filho M.C. (2001). Effects of soybean proteinase inhibitor on development, survival and reproductive potential of the sugarcane borer, diatraea saccharalis. Entomol. Exp. Appl..

[68] Pompermayer P., Falco M.C., Parra J.R.P., Silva-Filho M.C. (2003). Coupling diet quality and bowman-birk and kunitz-type soybean proteinase inhibitor effectiveness to *Diatraea saccharalis* development and mortality. Entomol. Exp. Appl..

[69] Zybailov B., Friso G., Kim J., Rudella A., Rodriguez V.R., Asakura Y., Sun Q., van Wijk K.J. (2009). Large scale comparative proteomics of a chloroplast clp protease mutant reveals folding stress, altered protein homeostasis, and feedback regulation of metabolism. Mol. Cell Proteom..

[70] Zheng B., Halperin T., Hruskova-Heidingsfeldova O., Adam Z., Clarke A.K. (2002). Characterization of chloroplast clp proteins in arabidopsis: Localization, tissue specificity and stress responses. Physiol. Plant.

[71] Porankiewicz J., Schelin J., Clarke A.K. (1998). The ATP-dependent Clp protease is essential for acclimation to UV-B and low temperature in the cyanobacterium *Synechococcus*. Mol. Microbiol..

[72] Abdeen A., Virgos A., Olivella E., Villanueva J., Aviles X., Gabarra R., Prat S. (2005). Multiple insect resistance in transgenic tomato plants over-expressing two families of plant proteinase inhibitors. Plant Mol. Biol..

[73] Maheswaran G., Pridmore L., Franz P., Anderson M.A. (2007). A proteinase inhibitor from *Nicotiana alata* inhibits the normal development of light-brown apple moth, *Epiphyas postvittana* in transgenic apple plants. Plant Cell Rep..

[74] Rose D., Schena M. (2000). Microfluidic technologies and instrumentation for printing DNA microarrays. Microarray Biochip Technology.

[75] Ross M.T., LaBrie S., McPherson J., Stanton V.P. (2001). Screening large-insert libraries by hybridization. Curr. Prot. Hum. Genet..

[76] Schummer M., Ng V.L.V., Baumgarner R.E., Nelson P.S., Schummer B., Bednarski D.W., Hassell L., Baldwin R.L., Karlan B.Y., Hood L. (1999). Comparative hybridization of an array of 21,500 ovarian cDNAs for the discovery of genes overexpressed in ovarian carcinomas. Gene.

[77] Vicentini R., Menossi M. (2007). Pipeline for macro- and microarray analyses. Braz. J. Med. Biol. Res..

[78] Sambrook J., Russel D.W. (2001). Molecular Cloning: A Laboratory Manual.

[79] Drummond R., Pinheiro A., Rocha C., Menossi M. (2005). ISER: Selection of differentially expressed genes from DNA array data by non-linear data transformations and local fitting. Bioinformatics.

[80] Pfaffl M.W. (2001). A new mathematical model for relative quantification in real-time RT-PCR. Nucleic Acid Res..

