# Supplementary material for: Sugarcane Serine Peptidase Inhibitors, Serine Peptidases, and Clp Protease System Subunits Associated with Sugarcane Borer (Diatraea saccharalis) Herbivory and Wounding
Source: Int J Mol Sci. 2016 Sep 1;17(9):1444. doi: 10.3390/ijms17091444 (PMC5037723; doi:10.3390/ijms17091444)
Supplement: Supplementary file 1 [file ijms-17-01444-s001.pdf]

Supplementary Materials: Sugarcane Serine Peptidase Inhibitors, Serine Peptidases, and Clp Protease System Subunits Associated with Sugarcane Borer (*Diatraea saccharalis*) Herbivory and Wounding

Ane H. Medeiros, Fabiana B. Mingossi, Renata O. Dias, Flávia P. Franco, Renato Vicentini, Marcia O. Mello, Daniel S. Moura and Marcio C. Silva-Filho

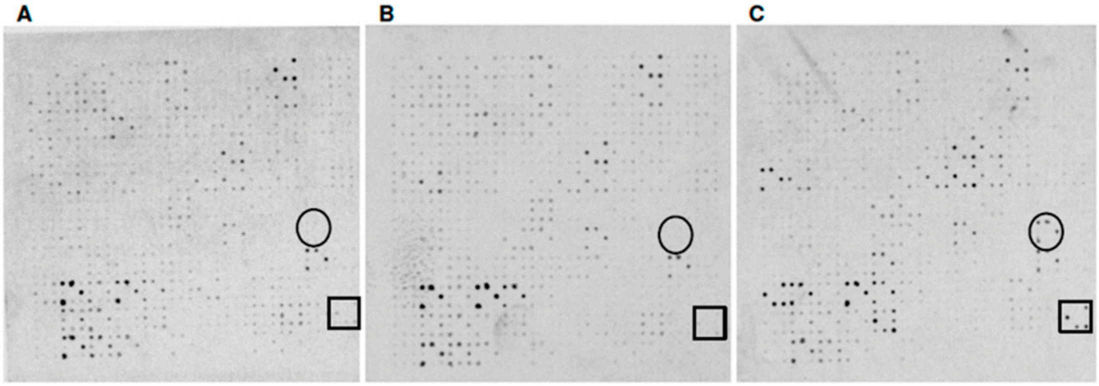

Figure S1. Hybridization signal of the filter membranes probed with ^{33}P cDNA populations derived from RNA extracted from leaves of sugarcane plants unattacked at 0 (zero) (**A**) and at 9 h time points (**B**) and attacked by *D. saccharalis* (**C**) at 9 h time point. The clones market with a circle and a square show marked differences in hybridization between treatments (refer to text for details). The hybridization signal was detected with *Storm 860 Phosphorimager* (Bio-Rad) at 50 μm resolution.

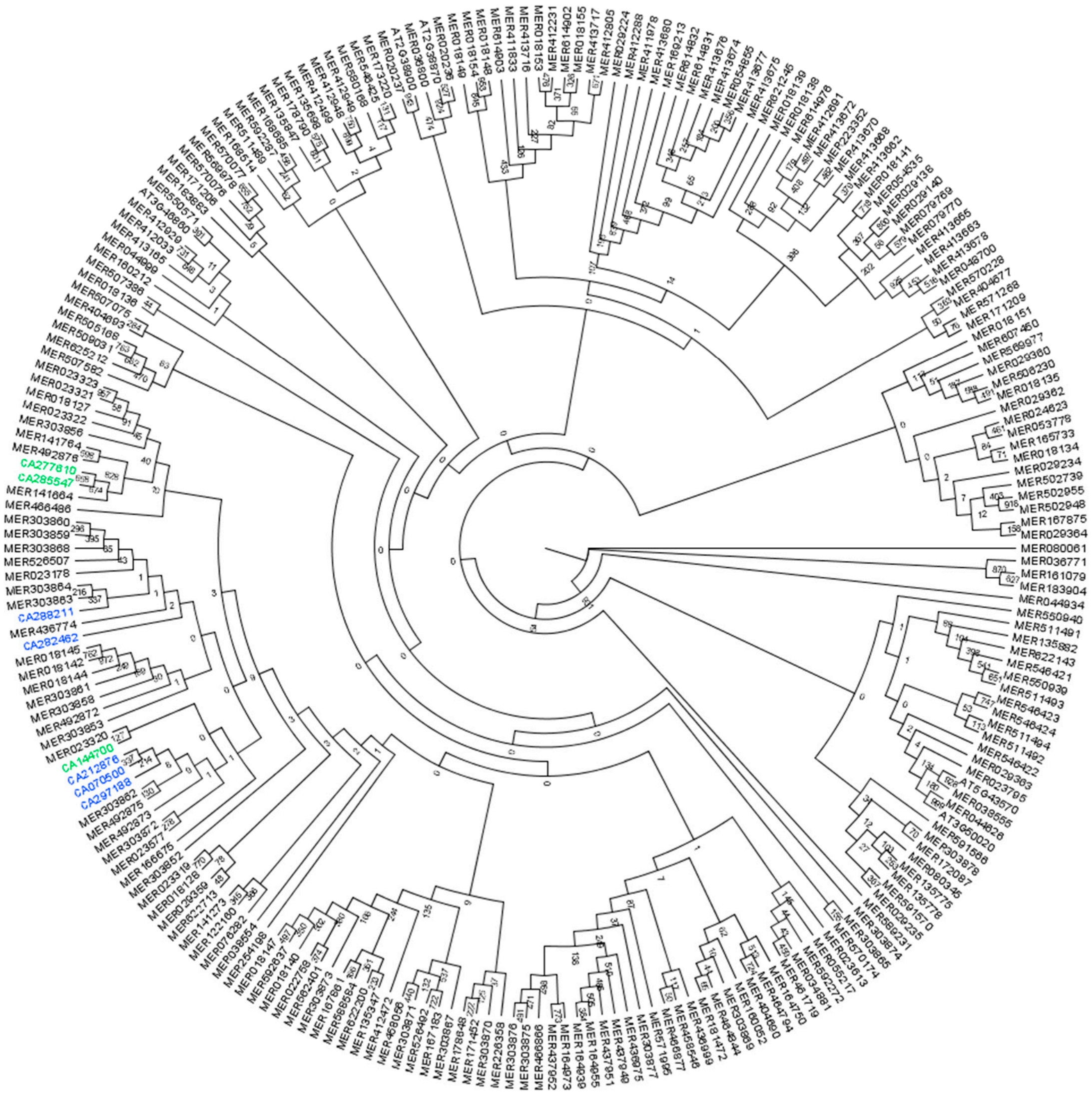

Figure S2. Maximum-likelihood phylogenetic proposition with protein sequences from the I13 peptidase inhibitor family. Induced and not-responsible genes from sugarcane to insect feeding are presented in blue and green, respectively. Except by sugarcane and *Arabidopsis*, all sequences were named according their accession numbers on the MEROPS database. The numbers in the nodes presents the bootstrap values for 1000 replicates.

Table S1. List of sugarcane ESTs present in the macroarray.

Accession List	GI List	Expression Profile	Observations
		**Peptidases**	
		**S10-Carboxypeptidase Y**	
CA113017.1	34966324	SacCPD-like2-Down	
CA149102.1	35050806	SacCPD-like3-Down	
CA064734.1	34916258		
CA064894.1	34916418		
CA071982.1	34924133		
CA080071.1	34932614		
CA084622.1	34937929		
CA097186.1	34950493		
CA099286.1	34952593		
CA102693.1	34956000		

Table S1. *Cont.*

Accession List	GI List	Expression Profile	Observations
CA107338.1	34960645		
CA114916.1	34968223		
CA118684.1	34971992		
CA121426.1	34974734		
CA130496.1	35013416		
CA133643.1	35019843		
CA134269.1	35021103		
CA136836.1	35026227		
CA137654.1	35027851		
CA137804.1	35028148		
CA138449.1	35029434		
CA146392.1	35045384		
CA147730.1	35048058		
CA150291.1	35053196		
CA150375.1	35053364		
CA152305.1	35057243		
CA154647.1	35061950		
CA154746.1	35062176		
CA157666.1	35068170		
CA166899.1	35086776		
CA170675.1	35094337		
CA173789.1	35100768		
CA173790.1	35100770		
CA182513.1	35118354		
CA189538.1	35132761		
CA197382.1	35227587		
CA197850.1	35228521		
CA201786.1	35236401		
CA211155.1	35255184		
CA214149.1	35261155		
CA217495.1	35267849		
CA219072.1	35271009		
CA223156.1	35279202		
CA223244.1	35279354		
CA260371.1	35950230		
CA260937.1	35951375		
CA281274.1	36010585		
CA285940.1	36027210		
		**S08-Subtilisin Carlsberg**	
CA094990.1	34948297	SacSub-like1-Down	
CA114161.1	34967468	SacSub-like2-Down	
CA114638.1	34967945	SacSub-like3-Down	
CA071485.1	34923636		
CA093030.1	34946337		
CA101463.1	34954770		
CA104634.1	34957941		
CA111239.1	34964546		
CA111889.1	34965196		
CA119540.1	34972848		
CA133894.1	35020344		
CA141062.1	35034662		
CA141064.1	35034665		
CA142393.1	35037349		

Table S1. *Cont.*

Accession List	GI List	Expression Profile	Observations
CA143066.1	35038684		
CA144000.1	35040540		
CA151385.1	35055383		
CA151610.1	35055835		
CA157640.1	35068118		
CA158691.1	35070254		
CA173567.1	35100301		
CA173767.1	35100724		
CA174056.1	35101319		
CA175309.1	35103899		
CA175948.1	35105158		
CA176493.1	35106283		
CA182320.1	35117971		
CA184434.1	35122199		
CA220022.1	35272885		
CA227201.1	35287366		
CA231626.1	35296353		
CA232779.1	35298624		
CA233816.1	35300737		
CA241444.1	35316063		
CA274786.1	35991621		
CA275973.1	35995003		
CA286426.1	36032147		
CA288984.1	36040024		
		**S01-Chymotrypsin**	
CA258670.1	35946811	SacChy-like-UP	
CA066138.1	34917662		
CA077204.1	34929476		
CA078375.1	34930647		
CA093912.1	34947219		
CA098045.1	34951352		
CA110765.1	34964072		
CA117747.1	34971055		
CA119574.1	34972882		
CA122266.1	34975574		
CA131057.1	35014660		
CA137905.1	35028353		
CA140307.1	35033170		
CA146851.1	35046300		
CA148171.1	35048941		
CA152098.1	35056812		
CA158463.1	35069793		
CA164966.1	35082903		
CA185269.1	35123867		
CA210210.1	35253285		
CA215958.1	35264725		
CA220175.1	35273215		
CA227937.1	35288950		
CA228604.1	35290282		
CA230885.1	35294868		
CA250521.1	35334264		
CA250875.1	35334976		
CA285697.1	36026512		

Table S1. *Cont.*

Accession List	GI List	Expression Profile	Observations
		**S09-Prolyl Oligopeptidase**	
CA096901.1	34950208		
CA112513.1	34965820		
CA119423.1	34972731		
CA119585.1	34972893		
CA124082.1	34977390		
CA124806.1	35002070		
CA135385.1	35023339		
CA137864.1	35028270		
CA147330.1	35047259		
CA147867.1	35048336		
CA151850.1	35056317		
CA155764.1	35064211		
CA161848.1	35076654		
CA300492.1	36074710		
		**S16-Lon-A Peptidase**	
CA113051.1	34966358		
CA130533.1	35013554		
CA152255.1	35057142		
CA153801.1	35060243		
CA171138.1	35095263		
CA175931.1	35105124		
CA176354.1	35105999		
CA236255.1	35305651		
		**S28-Lysosomal Pro-Xaa Carboxypeptidase**	
CA067463.1	34918987		
CA069602.1	34921126		
CA083873.1	34937184		
CA101244.1	34954551		
CA130602.1	35013721		
CA135512.1	35023593		
		**S54-Rhomboid-1**	
CA119754.1	34973062		
CA128957.1	35010352		
CA148478.1	35049556		
CA152909.1	35058452		
CA166487.1	35085947		
CA190014.1	35133757		
		**S26-Signal Peptidase I**	
CA093935.1	34947242		
CA111006.1	34964313		
CA129369.1	35011174		
CA284824.1	36023529		
		**S41-C-terminal Processing Peptidase-1**	
CA094899.1	34948206		
CA146320.1	35045237		
CA248375.1	35329949		
CA273646.1	35987493		
		**S33-Prolyl Aminopeptidase**	
CA111771.1	34965078		
CA143221.1	35039000		
		**S49-Signal Peptide Peptidase A**	
CA126570.1	35005589		
CA140468.1	35033491		

Table S1. *Cont.*

Accession List	GI List	Expression Profile	Observations
		**S59-Nucleoporin 145**	
CA123014.1	34976322		
CA127178.1	35006801		
		**Not Classified Peptidases**	
CA278685.1	36002999	SacCPD-like1-UP	
CA081360.1	34934308		
CA088102.1	34941409		
CA095246.1	34948553		
CA107226.1	34960533		
CA110047.1	34963354		
CA114317.1	34967624		
CA117007.1	34970302		
CA117207.1	34970514		
CA119381.1	34972689		
CA119499.1	34972807		
CA120052.1	34973360		
CA124832.1	35002122		
CA128959.1	35010356		
CA151956.1	35056529		
CA167091.1	35087162		
CA189368.1	35132409		
CA240268.1	35313701		
CA289713.1	36042407		
		**Clp Proteins**	
CA119497.1	34972805		ClpP1
CA119729.1	34973037		ClpP3
CA107353.1	34960660		ClpP4
CA074329.1	34926602		ClpP5
CA183086.1	35119501		ClpP5
CA282400.1	36014144		ClpP6
CA108609.1	34961916		ClpR1
CA108695.1	34962002		ClpR1
CA087622.1	34940929	SacClp-like2-Down	ClpR2
CA113615.1	34966922		ClpR4
CA164148.1	35081269	SacClp-like5-Down	ClpC1
CA126553.1	35005555	SacClp-like4-Down	ClpC1
CA119085.1	34972393		ClpC1
CA132637.1	35017831		ClpC1
CA124181.1	34977489		ClpC1
CA113004.1	34966311	SacClp-like3-Down	ClpC2
CA212375.1	35257605		ClpD
CA119462.1	34972770		ClpD
CA136349.1	35025255	SacClp-like1-UP	ClpD
CA145821.1	35044247		ClpD
CA194919.1	35222644		ClpD
CA147103.1	35046805		
		**Peptidase Inhibitors**	
		**I12-Bowman-Birk Inhibitor**	
CA090161.1	34943468		
CA095678.1	34948985		
CA121693.1	34975001		
CA143650.1	35039845		
CA146438.1	35045478		

Table S1. *Cont.*

Accession List	GI List	Expression Profile	Observations
CA151193.1	35055024		
CA188108.1	35129710		
CA232567.1	35298199		
CA261007.1	35951517	SacBBI2-UP	
CA266304.1	35965021	SacBBI3-UP	
CA272687.1	35984624	SacBBI4-UP	
CA273430.1	35986907		
CA276901.1	35997808		
CA285609.1	36026241		
**I13-Eglin C/Potato Peptidase Inhibitor I**			
CA070500.1	34922345	SacMPI-like1-UP	
CA212876.1	35258606	SacMPI-like2-UP	
CA282462.1	36014330	SacMPI-like3-UP	
CA288211.1	36037506	SacMPI-like4-UP	
CA297188.1	36065043	SacMPI-like5-UP	
CA144700.1	35041966		
CA277610.1	35999827		
CA285547.1	36026052		
**I04-α-1-Peptidase Inhibidor**			
CA135133.1	35022841		
CA151036.1	35054686		
CA179742.1	35112828		
**I03-Soybean Kunitz Trypsin Inhibitor**			
CA225401.1	35283725		
**Not Classified Peptidase Inhibitors**			
CA113558.1	34966865	SacBBI1-UP	
CA129230.1	35010896	SacCI-1B-like-UP	
CA124358.1	34977666		
**Housekeeping**			
CA124764.1	35001985		Actin
CA125181.1	35002825		Actin
CA146830.1	35046256		Actin
CA189477.1	35132625		Actin
CA117389.1	34970697		Actin
CA120082.1	34973390		Actin
CA146896.1	35046390		Polyubiquitin
CA093490.1	34946797		Polyubiquitin
CA116140.1	34969447		Polyubiquitin
CA116403.1	34969710		Polyubiquitin
CA119554.1	34972862		Glyceraldehyde-3-phosphate dehydrogenase
CA093039.1	34946346		Glyceraldehyde-3-phosphate dehydrogenase
CA171639.1	35096345		Glyceraldehyde-3-phosphate dehydrogenase
CA119610.1	34972918		Translational initiation factor elF-4A
CA190042.1	35133814		Eukaryotic initiation factor 4A
CA172318.1	35097704		Eukaryotic initiation factor 4A

Table S2. Sugarcane primer sequences used in real-time quantitative PCR.

Accession Number	Forward Primer (5′-3′)	Reverse Primer (5′-3′)
	**Sugarcane Up-regulated Clones**	
SacBBI1	GAGGTCGTCGCAGGTGTAGA	TGACAAACTCGAAGCCTCCT
SacBBI2	TGCACCAACTGCAACTTCTC	GTGTCGGTGCACTTGAACAT
SacBBI3	TGATCAGCTCTACTGAATCGTTG	GGTCAGCGGCATACATATAGAAG
SacBBI4	AACCCACAAGCACAATGACA	ACTCGGGCAAGATGTTCAAG
SacMPI-like1	GCTAGCTTCGGTTCCTTCCT	CAAACAACTGATCCCCTTTCA
SacMPI-like2	TGCATCGTCATCGATCAACT	ACCTTCTTGGCTTCCTCCAC
SacMPI-like3	GCCAAGAAGGTGATCCTCAA	CTGATCGATGAGCACGAAAG
SacMPI-like4	GGGTGACCGATGATTTTCTG	AACACACATCCGGAACCATT
SacMPI-like5	TGTAACCACCGATTTTGTGC	CACATCCATTATTCGGCAGAT
SacCI-1B-like	GATAAGCTAGCCGCTGATGC	GACCCATTATTCGGCAGATG
SacClp-like1	CCGCTTCCAGCCAGTATTAG	CATTGATGGCTTCCAATGTG
SacChy-like	CTCCAGGAGGTGATTTCTCG	TTGAGTGTTGTGGAGCTTGAA
SacCPD-like1	TTGTATGGACGCTCTGCTTG	TATGCCTGGCCTAGCAATTC
	**Sugarcane Down-regulated Clones**	
SacClp-like2	TGTTGGCAAGCATGCTGTAT	CACCAGCCGCAAGAATAAAT
SacClp-like3	CTTGCTCCGCAGAAGAAACT	CACAGGAGACTGGACACGAC
SacClp-like4	TAGTGCATTGGTGCCACAAC	GTGCAGCAGCAATTAAAGCA
SacClp-like5	CCTGCTCCAATCAGTGTGTG	CCTAATTGGAGAGCCTGGTG
SacCPD-like2	AGCTCTTCGAGCACTTCCTG	CGGTTGGCCAAAGTTGATT
SacCPD-like3	GGCGCTTATCTTGTCCTCCT	TCTCTCCCCTGGTCTCCTCT
SacSub-like1	TTCTGCCCATGACAGGGTAT	CTAGGCGACTCGAATCTTGG
SacSub-like2	CAAGACGAGGAAGGAATGGA	GGTCCGCAAGGTTTTAGCTT
SacSub-like3	GCTACATCAACTCCAGCTCCA	GTCCCCGACATGATGTTGTA
	**BBI Sugarcane Sequences**	
SacBBI5	CCTGGGTGGTGTCTTCTGAT	GCATCATGCATGGCTCTTTA
SacBBI6	AGAAGTGCGTCAAGCAAGGT	ACGAAGCTCGAGAGAAGCAT
SacBBI7	TTGACGCACTTCTTGCACTC	CAACAAACTTGGCATTGTGG
SacBBI8	TGCTTCTCTCGAGCTTGTCA	AAAGCACAACGCGAACTACA
SacBBI9	CTGACGCACTTCTTGCACTC	CATCGTCACTCTGGCTGTTC
SacBBI10	CATGCAAGCCATGTTGAGTT	TAGCATCTGCAAAGGGACAA
SacBBI11	AGCTTCGTCGGAGAAGGAAT	TAGCATCTGCAGAGGGAACA
SacBBI12	GACCGCGTTTCCAACTTCT	GGTTGCTCGCTCATCAGATT
SacBBI13	GCAGGGACTGCGTCAAGT	CTGGGTGGGTAAAGGTTGG
SacBBI14	CGGACATCCTCGTCAAATTC	GGCCTAGCCAAAGCTTATATTG
	**Sugarcane Reference Genes**	
GAPDH	TTTGAATGGCAAGCTCACTG	GGTGGAAACCAAATCCTCCT
β-actin	TCTACGGCAACATTGTGCTC	TTGATCTTCATGCTGCTTGG
